# Development and evaluation of immunogenicity and protective efficacy of two recombinant attenuated newcastle disease viruses expressing the VP2 protein of infectious bursal disease virus

**DOI:** 10.1016/j.psj.2025.105253

**Published:** 2025-05-08

**Authors:** Xiaolong Fan, Bolong Li, Lijing Zhu, Yuanmeng Shen, Han Zhou, Guojie Ding, Jiaxuan Li, Yanping Jiang, Wen Cui, Lijie Tang, Shuo Jia, Yijing Li

**Affiliations:** a College of Veterinary Medicine, Northeast Agricultural University, Harbin 150030, China; b Harbin Vaccine science Biotechnology Co., Ltd., Harbin 150030, China; c Heilongjiang Key Laboratory for Animal Disease Control and Pharmaceutical Development, Harbin 150030, China

**Keywords:** Newcastle disease virus, Infectious bursal disease virus, recombinant attenuated virus, VP2 protein, immunogenicity

## Abstract

Newcastle disease (**ND**) and Infectious bursal disease (**IBD**) are highly contagious and economically significant viral diseases affecting poultry worldwide. Despite widespread vaccination, current vaccines often require multiple doses and provide limited protection against emerging strains. This study developed and evaluated bivalent vaccines based on recombinant Newcastle disease virus (**NDV**) expressing the VP2 protein of a novel variant Infectious bursal disease virus (**IBDV**), SHG19 strain. To enhance immunogenicity and antigenic compatibility, two recombinant NDVs were generated: rHV, using a modified genotype VII HEB strain backbone, and rLHV, using the lentogenic LaSota backbone incorporating HEB F and HN genes. Protective efficacy was evaluated in specific pathogen-free (**SPF**) chickens through immunization followed by challenges with both NDV and IBDV. Results demonstrated robust humoral immune responses, significant reductions in viral shedding, and effective dual protection against both pathogens. These findings highlight the potential of NDV-based bivalent vaccines to streamline vaccination protocols and offer enhanced protection against circulating NDV and IBDV strains.

## Introduction

Newcastle disease (**ND**) and Infectious bursal disease (**IBD**) are major viral diseases affecting the global poultry industry (Myint et al., 2021; Charkhkar et al., 2024). Newcastle disease virus (**NDV**), a member of the *Paramyxoviridae* family, causes severe respiratory and neurological symptoms and can result in high mortality rates in unvaccinated flocks (Rehman et al., 2018). Infectious bursal disease virus (**IBDV**), a member of the *Birnaviridae* family, targets the bursa of Fabricius, causing immunosuppression and increasing susceptibility to secondary infections (Franciosini and Davidson, 2022; Trapp and Rautenschlein, 2022).

Vaccination is the primary control strategy for ND and IBD. However, current vaccines, including live-attenuated and inactivated forms, often require multiple doses, leading to increased labor costs, handling stress in chickens, and potential interference with other vaccinations (Courtillon et al., 2022). These limitations underscore the need for improved vaccine strategies, such as bivalent vaccines, providing simultaneous protection against both ND and IBD.

NDV is a promising vector for vaccine development due to its well-characterized genome, ability to stably express foreign genes, and capacity to induce strong immune responses (Kim and Samal, 2016; Hu et al., 2020; de Swart and Belov, 2023). Reverse genetics systems have facilitated the creation of recombinant NDV vaccines expressing protective antigens from other pathogens. These vaccines can stimulate both humoral and cellular immunity, providing protection against NDV and other pathogens when engineered to express their antigens (He et al., 2023; Lee et al., 2023; Tan et al., 2024; Zeng et al., 2024; Liu et al., 2025), including viral antigens from non-avian species (Sun et al., 2021; Dey et al., 2024; Murr et al., 2024; Soliman et al., 2024).

The epidemiology of IBDV has shifted significantly, with the emergence of novel variant infectious bursal disease virus (**nVarIBDV**) strains across various regions, including China, Japan, South Korea, and Malaysia (Fan et al., 2019; Aliyu et al., 2021; Myint et al., 2021; Thai et al., 2022). These nVarIBDV strains evade existing vaccine-induced immunity, causing immunosuppression, severe bursal damage, and substantial economic losses (Fan et al., 2020; Li et al., 2023b). The VP2 protein, the major capsid protein of IBDV, contains key neutralizing epitopes and is a well-established target for vaccine development (Li et al., 2020, 2023a; Soleymani et al., 2023). Therefore, VP2 is frequently used as an immunogenic antigen in recombinant viral-vectored vaccines (Shah et al., 2022; Eldaghayes et al., 2023; Soleymani et al., 2023; Liu et al., 2025).

This study aimed to develop and assess recombinant NDV-based vaccines expressing the VP2 protein derived from the emergent IBDV SHG19 strain. We constructed two stable recombinant NDVs expressing the IBDV SHG19 VP2, evaluated their immunogenicity in chickens, and assessed their protective efficacy against challenges with both NDV and two distinct IBDV strains: a novel variant strain (GF6) and a commonly used challenge strain (BC6/85). This approach provides a potential solution to simplify vaccination programs while enhancing protection against circulating IBDV variants.

## Materials and methods

### Cell, viruses, vaccines, plasmids, and animals

BHK-21 cells expressing T7 RNA polymerase (BHK-T7 cells) were cultured in Dulbecco's Modified Eagle Medium (DMEM; Gibco) supplemented with 10 % fetal bovine serum (FBS; Gibco, Gaithersburg, MD) at 37 °C and 5 % CO₂. NDV strains, LaSota and genotype VII HEB, and the pCI eukaryotic expression vector used for cloning and expression were available in our laboratory. The IBDV challenge strains, GF6 (a novel variant IBDV strain) and BC6/85 (a standard challenge strain commonly used for evaluating IBDV vaccine efficacy), and commercial NDV and IBDV vaccines were obtained from Harbin Vaccine science Biotechnology Co., Ltd. (Harbin, China). The IBDV VP2 gene, based on the SHG19 sequence (GenBank accession no. MN393076), was synthesized by Jilin Kumei Biotechnology Co., Ltd. (Harbin, China). Specific-pathogen-free (**SPF**) chickens and embryonated chicken eggs were purchased from the Experimental Animal Center of the Harbin Veterinary Research Institute (**HVRI**), Chinese Academy of Agricultural Sciences (**CAAS**), and were housed in isolators with negative-pressure-filtered air. All animal experiments were conducted in compliance with the Guide for the Care and Use of Agricultural Animals in Agriculture Research and Teaching of Northeast Agricultural University.

### Plasmid construction

Two recombinant full-length plasmids, derived from the LaSota and genotype VII HEB strains, were constructed. The first backbone, pCI-aH, was derived from the genotype VII HEB strain by introducing nucleotide mutations into the F gene to modify the cleavage site to match the lentogenic LaSota sequence. The second backbone, pCI-L-H(aF/HN), was derived from the LaSota strain by replacing the endogenous F and HN genes with their HEB strain counterparts (using the HEB F gene containing the aforementioned cleavage site mutations).

To generate the final bivalent vaccine constructs, the IBDV VP2 gene (derived from the SHG19 strain) was inserted into these two modified NDV backbones, pCI-aH and pCI-L-H(aF/HN). An expression cassette containing the VP2 gene, flanked by appropriate restriction sites (*Pme* I) and incorporating necessary signals including NDV gene end and gene start sequences and a Kozak sequence, was designed as detailed in Supplementary Figure 1. This cassette was inserted between the P and M genes using the ClonExpress Ultra One Step Cloning Kit (Vazyme, Nanjing, China) within the context of the pCI eukaryotic expression vector system. The sequences of the resulting full-length recombinant plasmids, designated pCI-aH-VP2 and pCI-L-H(aF/HN)-VP2, were confirmed by sequencing.

Helper plasmids were constructed for rescuing recombinant viruses. One set expressed the NP, P, and L proteins of the genotype VII HEB strain, and the other expressed the corresponding proteins of the LaSota strain. These helper plasmids, also expressed in the pCI vector, were verified by restriction enzyme digestion (*Xba* I; New England Biolabs, Ipswich, MA) and sequencing. The specific primer sequences used to amplify all necessary gene fragments for constructing both the full-length recombinant plasmids and the helper plasmids are listed in Supplementary Table 1.

### Recombinant virus rescue and hemagglutination assay

When BHK-T7 cells reached 80 % to 90 % confluence, the full-length plasmids and corresponding helper plasmids (HEB or LaSota; NP, P, L at a 4:2:1:1 mass ratio) were co-transfected into cells using Lipofectamine 3000 (Thermo Fisher Scientific, Waltham, MA). Following transfection, the medium was replaced after 24 h with serum-free medium supplemented with TPCK-treated trypsin (Sigma-Aldrich, St. Louis, MO). At 72 h post-transfection, the cell culture supernatant was harvested and inoculated into 9-day-old SPF embryonated chicken eggs. The allantoic fluid was harvested after 72 h of incubation at 37 °C, and passaged three times in 9-day-old SPF embryonated chicken eggs.

Virus titers were determined by hemagglutination (**HA**) assay. Briefly, 25 μL of allantoic fluid was serially two-fold diluted in PBS, mixed with an equal volume of 1 % chicken red blood cell (**RBC**) suspension, and incubated at room temperature for 30-45 min. The HA titer was the highest dilution with no RBC streaming.

### Confirmation of VP2 protein expression by western blot (WB)

VP2 protein expression in the rescued recombinant viruses was confirmed by Western blot analysis. BHK-T7 cells were infected with rHV or rLHV at an MOI of 1. Cells were harvested at 24 h post-infection, washed with PBS, lysed in radioimmunoprecipitation assay (**RIPA**) lysis buffer (Beyotime, Shanghai, China) containing protease inhibitors for 30 min on ice. Lysates were centrifuged at 12,000 g for 10 min at 4 °C, and the supernatants were collected. The supernatants were mixed with SDS-PAGE sample buffer, boiled for 10 min, and subjected to SDS-PAGE on a 10 % polyacrylamide gel. The separated proteins were transferred to a polyvinylidene difluoride (**PVDF**) membrane (Millipore, Milford, MA). The membrane was blocked with 5 % non-fat milk in PBST (0.1 % Tween-20) for 1 h at room temperature and then incubated overnight at 4 °C with a mouse anti-VP2 polyclonal antibody (1:200 dilution; produced in-house). The membrane was washed three times with PBST and then incubated with horseradish peroxidase (**HRP**)-conjugated goat anti-mouse IgG (1:5,000 dilution; ZSGB-Bio, Beijing, China) for 1 h at 37 °C. Protein bands were visualized using an enhanced chemiluminescence (**ECL**) detection system (ChemiScope 3100 Mini; Clinx Science Instruments Co., Ltd., Shanghai, China).

### Confirmation of VP2 protein expression by immunofluorescence assay (IFA)

IBDV VP2 protein expression in the rescued recombinant viruses was further confirmed by IFA. BHK-T7 cells, seeded in 6-well plates, were infected with either rHV or rLHV at an MOI of 1. After incubation at 37 °C for 24 h, the cells were washed with PBS, fixed with 4 % paraformaldehyde for 15 min at room temperature, and permeabilized with 0.1 % Triton X-100 in PBS for 10 min. The cells were blocked with 3 % BSA in PBS for 1 h at room temperature. The cells were incubated overnight at 4 °C with primary antibodies: chicken anti-NDV polyclonal serum (1:200 dilution; produced in-house) and mouse anti-VP2 polyclonal antibody (1:200 dilution). After three washes with PBS, the cells were incubated with secondary antibodies: fluorescein isothiocyanate (**FITC**)-conjugated goat anti-chicken IgG (1:100 dilution; Bioss, Beijing, China) and tetramethylrhodamine (**TRITC**)-conjugated goat anti-mouse IgG (1:100 dilution; ZSGB-Bio, Beijing, China) for 1 h at 37 °C in the dark. After three washes with PBS, fluorescence was visualized using a fluorescent cell imager (ZOE; Bio-Rad Laboratories, Inc., Hercules, CA).

### Experimental design, immunization, and challenge

The study included 13 groups, each containing six SPF chickens. This sample size of six chickens per group is consistent with common practices in poultry vaccine efficacy studies (Lee et al., 2022). The groups were organized as follows (Table 1):

**Table 1:** Experimental groups and treatments.

Group	Vaccine	Challenge
rHV + NDV	rHV	genotype VII NDV (HEB strain)
rHV + GF6	rHV	nVarIBDV (GF6 strain)
rHV + BC6/85	rHV	IBDV (BC6/85 strain)
rLHV + NDV	rLHV	genotype VII NDV (HEB strain)
rLHV + GF6	rLHV	nVarIBDV (GF6 strain)
rLHV + BC6/85	rLHV	IBDV (BC6/85 strain)
Commercial NDV vaccine + NDV	Commercial NDV vaccine	genotype VII NDV (HEB strain)
Commercial IBDV vaccine + GF6	Commercial IBDV vaccine	nVarIBDV (GF6 strain)
Commercial IBDV vaccine + BC6/85	Commercial IBDV vaccine	IBDV (BC6/85 strain)
NDV HEB control	PBS	genotype VII NDV (HEB strain)
IBDV GF6 control	PBS	nVarIBDV (GF6 strain)
IBDV BC6/85 control	PBS	IBDV (BC6/85 strain)
PBS control	PBS	PBS

Seven-day-old chickens were assigned to their respective treatment groups. The rHV and rLHV groups were immunized with 10^6^ EID_50_ of the respective recombinant virus via the oculonasal route. Chickens in the commercial vaccine groups were immunized according to the manufacturers' instructions via the same route. At 21 days post-immunization (**dpi**), chickens were challenged oculonasally with either the NDV genotype VII HEB strain (10^5^ ELD_50_ per chicken) or one of the IBDV strains (GF6 or BC6/85; 100 BID_50_ per chicken). Clinical signs and mortality were monitored daily for 7 days post-challenge (**dpc**). Surviving chickens were euthanized at the end of the observation period (7 dpc).

### Serological analysis

Serum samples were collected at 7, 14, and 21 dpi to determine NDV-specific antibody titers by hemagglutination inhibition (**HI**) assay. Two-fold serial dilutions of serum samples were incubated with 4 hemagglutinating units (**HAU**) of NDV antigen for 30 min at room temperature, followed by the addition of 1 % chicken RBC suspension. Plates were incubated for 30-45 min at room temperature, the HI titer was the highest dilution of serum causing complete inhibition of 4 HAU of antigen.

IBDV VP2-specific antibody titers were determined by enzyme-linked immunosorbent assay (**ELISA**). Microplates were coated with purified IBDV VP2 protein as antigen. Serum samples were added to the wells, and subsequent steps involving sample incubation, washing, addition of appropriate enzyme conjugates, and substrate development were performed according to the manufacturer’s guidelines for the commercial kit (Meimian Industrial Co., Ltd., Jiangsu, China).

### Virological analysis

For NDV shedding analysis, oropharyngeal and cloacal swabs collected on days 1, 3, 5, and 7 dpc were processed in PBS, centrifuged, and the supernatant was inoculated into 9-day-old SPF embryonated chicken eggs. Allantoic fluid harvested after 72 h incubation was tested by HA assay.

For IBDV shedding analysis, cloacal swabs collected daily for 7 dpc underwent RNA extraction using TRNzol Universal Reagent (TIANGEN, Beijing, China) and reverse transcription using TransScript One-Step gDNA Removal and cDNA Synthesis SuperMix (TransGen Biotech, Beijing, China). PCR was performed on the resulting cDNA using specific primers for the challenge strain (GF6-F/R or BC6/85-F/R; Supplementary Table 2).

### Histopathological analysis

Necropsies were performed on a subset of randomly selected chickens (three per group) at 3 dpc to assess gross pathological changes in the trachea, lungs, spleen, kidneys, proventriculus, duodenum, and bursa of Fabricius. Samples of these organs were collected during necropsy, fixed in 4 % paraformaldehyde solution for at least 24 h, processed routinely for paraffin embedding, sectioned, and stained with hematoxylin and eosin (H&E) for histopathological examination.

### Viral load analysis

Approximately 0.1 g of specific tissues were collected for viral load quantification. For NDV-challenged groups, the heart, liver, spleen, lung, kidney, thymus, bursa of Fabricius, trachea, and duodenum were analyzed. For IBDV-challenged groups, the bursa of Fabricius was analyzed. Total RNA extraction and reverse transcription to cDNA were performed using the same reagents and protocols as described above. Real-time quantitative PCR (**RT-qPCR**) was then conducted using cDNA as a template and F488 SYBR qPCR Mix (Nachuan Biotechnology Co., Ltd., Harbin, China) on a CFX96 real-time system (Bio-Rad, Hercules, CA). Specific primers for quantifying NDV HEB, IBDV GF6, and IBDV BC6/85 are listed in Supplementary Table 2.

### Survival analysis

Post-challenge survival was monitored daily for 7 days, and survival rates were calculated for each group to assess protective efficacy.

### Statistical analysis

The data were analyzed using GraphPad Prism (version 9.0; GraphPad Software, San Diego, CA). Statistical significance was determined using Student's t-test or ANOVA, followed by Duncan's new multiple range test for comparisons between treatments and controls. Differences were considered statistically significant at *P* < 0.05. Significance levels in figures and tables are indicated as follows: *P < 0.05, **P < 0.01, and ***P < 0.001.

## Results

### Construction and validation of recombinant plasmids

**Fig. 1:**
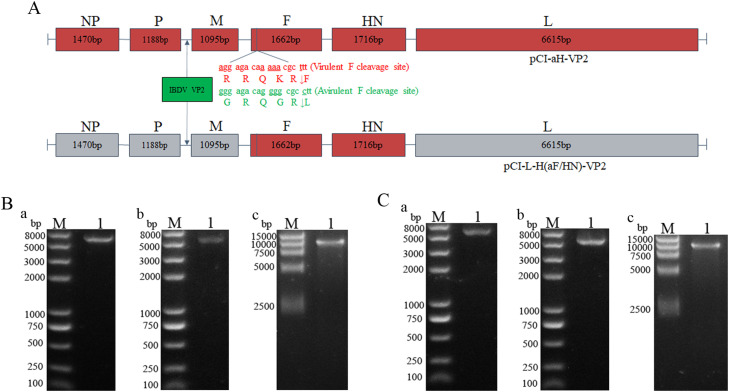
Construction and validation of recombinant plasmids. (A) Schematic diagrams illustrating the genomic organization of the final recombinant NDV full-length plasmids pCI-aH-VP2 and pCI-L-H(aF/HN)-VP2, showing the insertion of the IBDV VP2 gene between the P and M genes and indicating key backbone modifications (F gene mutation or F/HN gene replacement). (B and C) Agarose gel electrophoresis confirming the integrity of constructed helper plasmids following *Xba* I digestion. Lanes M: DNA marker; Lane 1: *Xba* I digested plasmid. (B) HEB-derived helper plasmids: (a) pCI-H-NP; (b) pCI-H-P; (c) pCI-H-L. (C) LaSota-derived helper plasmids: (a) pCI-L-NP; (b) pCI-L-P; (c) pCI-L-L.

Schematic representations of the constructed recombinant full-length plasmids, pCI-aH-VP2 and pCI-L-H(aF/HN)-VP2, are shown in Fig. 1A. These plasmids feature the IBDV VP2 gene inserted between the NDV P and M genes within modified backbones. The pCI-aH-VP2 plasmid incorporated nucleotide mutations in the F gene resulting in a modified cleavage site, while the pCI-L-H(aF/HN)-VP2 plasmid contained F and HN genes replaced with HEB strain counterparts (incorporating the same F gene cleavage site mutations). Sequence analysis confirmed that both full-length plasmids matched the designed sequences.

Two sets of helper plasmids (detailed maps in Supplementary Figure 2) were also constructed and validated: pCI-H-NP, pCI-H-P, and pCI-H-L (expressing HEB strain NP, P, and L proteins); and pCI-L-NP, pCI-L-P, and pCI-L-L (expressing LaSota strain NP, P, and L proteins). Restriction enzyme digestion (*Xba* I) and sequencing confirmed the integrity of both helper plasmid sets. As shown in Fig. 1B and 1C, *Xba* I digestion of the helper plasmids yielded fragments of approximately 5474 bp, 5192 bp, and 10619 bp, consistent with the expected sizes, respectively. These results confirmed the successful construction of both the recombinant full-length and helper plasmids, enabling subsequent virus rescue.

### Rescue of recombinant viruses and confirmation of hemagglutination activity

**Fig. 2:**
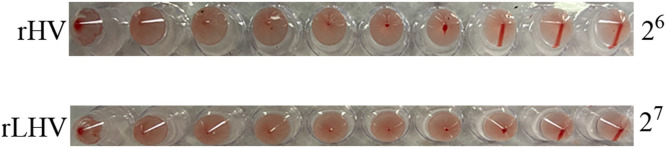
Confirmation of recombinant virus rescue by hemagglutination (**HA**) assay following propagation in embryonated eggs. Allantoic fluid, harvested after three serial passages in 9-day-old SPF embryonated chicken eggs, was subjected to HA assay. (A) rHV exhibited a titer of 2^6^ HA units. (B) rLHV exhibited a titer of 2^7^ HA units.

Following co-transfection of the recombinant NDV full-length plasmids (pCI-aH-VP2 and pCI-L-H(aF/HN)-VP2) and corresponding helper plasmids (expressing HEB or LaSota NP, P, and L proteins) into BHK-T7 cells, virus rescue was confirmed by HA assay (Fig. 2). Supernatants from transfected cells were inoculated into 9-day-old SPF embryonated chicken eggs. Following three serial passages in eggs, HA assays performed on the harvested allantoic fluid revealed titers of 2⁶ and 2⁷ HA units for rHV and rLHV, respectively. These HA titers indicate the successful rescue of functional, HA-active, and replication-competent recombinant viruses.

### Confirmation of VP2 protein expression in recombinant virus-infected cells

**Fig. 3:**
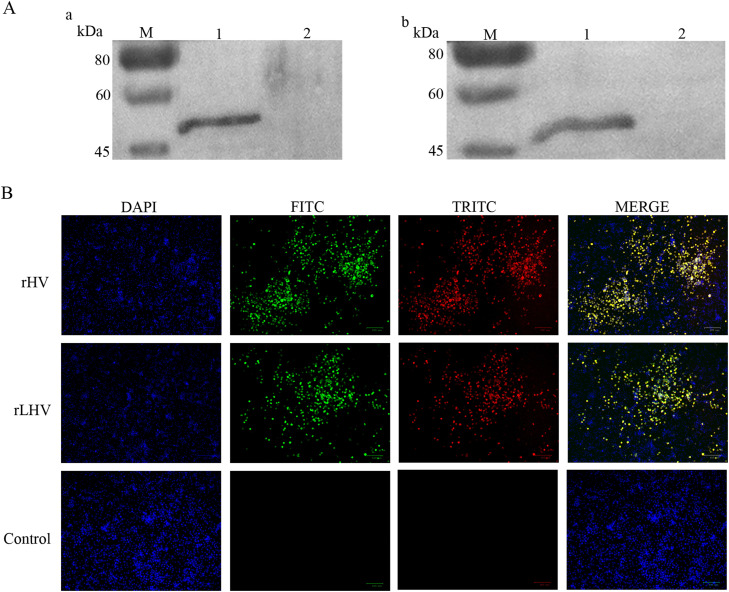
Confirmation of VP2 protein expression by Western blot and immunofluorescence assay. (A) Western blot analysis of BHK-T7 cell lysates using a mouse anti-VP2 polyclonal antibody. Lane M: protein marker. Lane 1 shows lysate from infected cells; Lane 2 shows lysate from uninfected control cells for: (a) rHV infection; (b) rLHV infection. (B) IFA of BHK-T7 cells infected with rHV (top row), rLHV (middle row), or mock-infected (Control, bottom row) at 24 h post-infection. Cells were co-stained with chicken anti-NDV serum (detected by FITC, green) and mouse anti-VP2 antibody (detected by TRITC, red). Nuclei were counterstained with DAPI (blue). Merged images are shown in the right column. Scale bars = 100 μm.

As shown in Fig. 3A, Western blot analysis detected a distinct band at approximately 50 kDa, corresponding to the expected molecular weight of VP2. This band was observed in lysates from cells infected with rHV (Fig. 3A, panel a, lane 1) and rLHV (Fig. 3A, panel b, lane 1), but not in the corresponding uninfected control lysates (Fig. 3A, panel a, lane 2 and panel b, lane 2).

Immunofluorescence assay (**IFA**) further visualized VP2 protein expression in infected BHK-T7 cells (Fig. 3B). Cells infected with rHV or rLHV showed green fluorescence (FITC) when stained with chicken anti-NDV serum, confirming NDV infection, and strong red fluorescence (TRITC) when stained with mouse anti-VP2 antibody, indicating VP2 expression. Merged images demonstrated co-localization (yellow fluorescence) of NDV and VP2 proteins in cells infected with rHV or rLHV, whereas control cells showed only background DAPI staining.

### Humoral antibody responses post-immunization

**Fig. 4:**
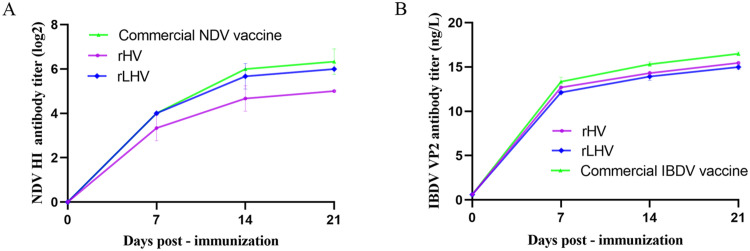
Humoral antibody responses in chickens following immunization. Serum samples collected at 7, 14, and 21 days post-immunization (**dpi**) were analyzed. (A) NDV-specific antibody titers determined by hemagglutination inhibition (HI) assay, expressed as log₂ values. (B) IBDV VP2-specific antibody titers determined by ELISA, expressed as ng/L. Data points represent the mean ± SEM.

Serum samples collected at 7, 14, and 21 days post-immunization (**dpi**) were assayed for NDV-specific hemagglutination inhibition (**HI**) antibodies and IBDV VP2-specific ELISA antibodies (Fig. 4). Both recombinant viruses, rHV and rLHV, induced increasing antibody titers over time. At 21 dpi, the mean NDV HI antibody titers were approximately 2^5^ for rHV, 2^6^ for rLHV, and 2^6.3^ for the commercial NDV vaccine group (Fig. 4A). At the same time point (21 dpi), mean IBDV VP2-specific antibody titers were approximately 15.45 ng/L for rHV, 14.99 ng/L for rLHV, and 16.49 ng/L for the commercial IBDV vaccine group (Fig. 4B).

### Survival rates post-challenge

**Fig. 5:**
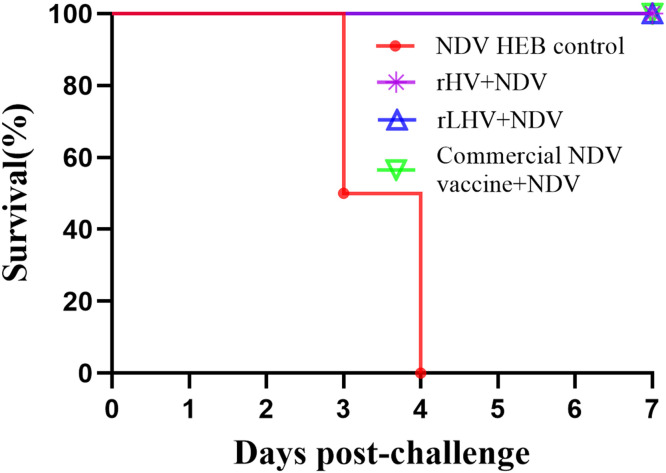
Survival curves of chickens following NDV challenge. The graph displays the percentage survival over time (days post-challenge, **dpc**) for each experimental group.

The protective efficacy against NDV challenge was assessed by monitoring survival rates for 7 days post-challenge (Fig. 5). Following challenge with the NDV HEB strain, chickens in the unvaccinated control group began showing mortality at 3 days post-challenge (**dpc**), with 100 % mortality observed by 4 dpc (0 % survival). In contrast, all chickens vaccinated with rHV, rLHV, or the commercial NDV vaccine survived the challenge (100 % survival) throughout the 7-day observation period. No mortality was observed in any group following IBDV challenge.

### Reduction of viral shedding in vaccinated groups

Vaccination significantly reduced viral shedding compared to unvaccinated controls following challenge. For the NDV challenge, shedding from oropharyngeal and cloacal swabs, assessed by virus isolation, was widespread and persistent in the unvaccinated control group until succumbing to infection, whereas shedding was markedly reduced or absent in the vaccinated groups (Table 2). Specifically, no virus was detected at any time point in swabs from rHV-immunized chickens, while only low-level, transient shedding (primarily at 3 dpc) was observed in the rLHV and commercial NDV vaccine groups. In the case of IBDV challenges, the duration of cloacal shedding detected by PCR was notably shorter in all vaccinated groups (rHV, rLHV, commercial IBDV vaccine) compared to the unvaccinated controls challenged with either GF6 (Table 3) or BC6/85 (Table 4).

**Table 2:** Shedding of challenged NDV HEB strain in oropharyngeal (**OP**)and cloacal (**CL**)swabs.

Group	Days post challenge (dpc)							
	1dpc		3dpc		5dpc		7dpc	
	OP	CL	OP	CL	OP	CL	OP	CL
rHV + NDV	0/6	0/6	0/6	0/6	0/3	0/3	0/3	0/3
rLHV + NDV	0/6	0/6	0/6	1/6	0/3	0/3	0/3	0/3
Commercial NDV vaccine + NDV	0/6	0/6	1/6	3/6	0/3	0/3	0/3	0/3
NDV HEB control	6/6	6/6	6/6	6/6	-	-	-	-

Data in the OP and CL swab columns represent the number of positive chickens out of the total number tested (positive/total) at the indicated days post-challenge (dpc).

**Table 3:** Shedding of challenged IBDV GF6 strain in cloacal (**CL**) swabs.

Group	Days post challenge (dpc)						
	1dpc	2dpc	3dpc	4dpc	5dpc	6dpc	7dpc
	CL	CL	CL	CL	CL	CL	CL
rHV+ GF6	0/6	1/6	2/6	0/3	0/3	0/3	0/3
rLHV+ GF6	0/6	1/6	1/6	0/3	0/3	0/3	0/3
Commercial IBDV vaccine + GF6	1/6	1/6	2/6	0/3	0/3	0/3	0/3
IBDV GF6 control	3/6	6/6	6/6	2/3	2/3	1/3	1/3

Data represent the number of positive chickens out of the total number tested (positive/total) at the indicated days post-challenge (**dpc**).

**Table 4:** Shedding of challenged IBDV BC6/85 strain in cloacal (**CL**) swabs.

Group	Days post challenge (dpc)						
	1dpc	2dpc	3dpc	4dpc	5dpc	6dpc	7dpc
	CL	CL	CL	CL	CL	CL	CL
rHV+ BC6/85	1/6	2/6	0/6	0/3	0/3	0/3	0/3
rLHV+ BC6/85	0/6	2/6	0/6	0/3	0/3	0/3	0/3
Commercial IBDV vaccine + BC6/85	1/6	2/6	4/6	3/3	0/3	0/3	0/3
IBDV BC6/85 control	3/6	6/6	6/6	3/3	1/3	0/3	0/3

Data represent the number of positive chickens out of the total number tested (positive/total) at the indicated days post-challenge (**dpc**).

### Protection against virus-induced pathology

**Fig. 6:**
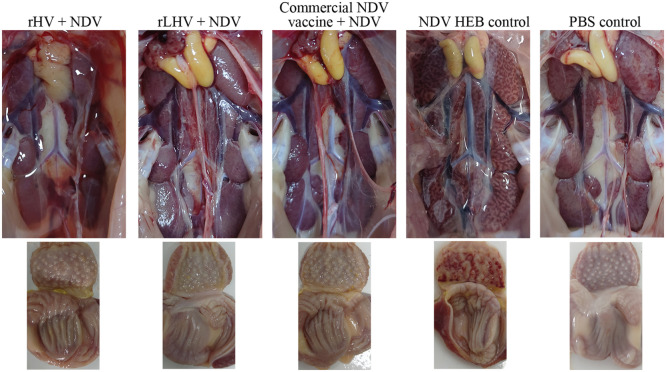
Gross pathological changes in kidneys and proventriculus following NDV challenge. Representative images display kidneys (top row) and proventriculus (bottom row) from chickens in the indicated experimental groups at 3 days post-challenge (**dpc**). Labels indicate the treatment/challenge group.

**Fig. 7:**
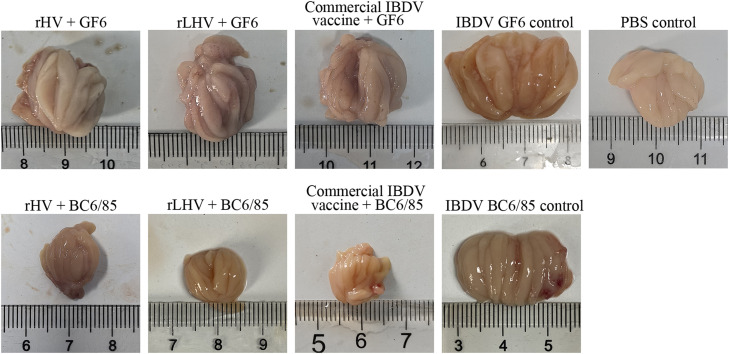
Gross pathological changes in the bursa of Fabricius following IBDV challenge. Representative images show bursae collected from chickens in the indicated experimental groups at 3 days post-challenge following challenge with either IBDV GF6 (top row of challenge groups) or IBDV BC6/85 (bottom row of challenge groups). Arrows indicate representative lesions such as petechial (black arrow) or extensive (red arrow) hemorrhages observed primarily in control groups.

At 3 days post-challenge (**dpc**), chickens in the vaccinated groups (rHV, rLHV, respective commercial vaccines) and the PBS control group displayed minimal to no gross lesions. Following NDV challenge, unvaccinated challenge controls suffered severe kidney lesions and extensive proventricular hemorrhages (Fig. 6). Similarly, after IBDV challenge, unvaccinated challenge controls exhibited characteristic bursal pathology, specifically petechial hemorrhages (GF6 strain) or extensive hemorrhages (BC6/85 strain) (Fig. 7).

**Fig. 8:**
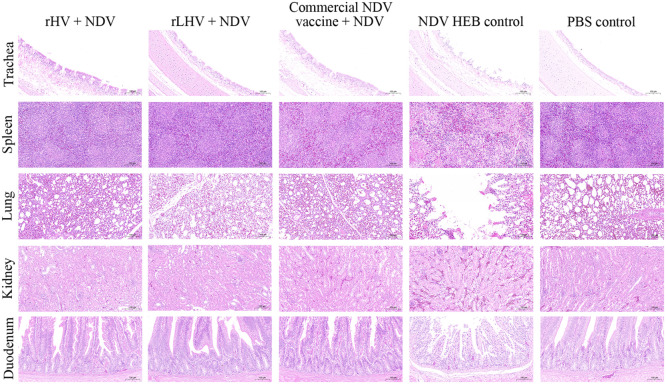
Histopathological changes in tissues following NDV challenge. Representative images of hematoxylin and eosin (**H&E**)-stained tissue sections (trachea, spleen, lung, kidney, duodenum) from chickens in the indicated experimental groups at 3 days post-challenge. Scale bar = 100 μm.

**Fig. 9:**
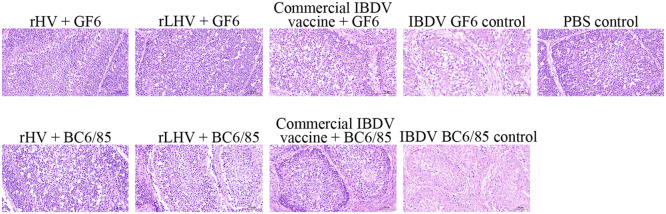
Histopathological changes in the bursa of Fabricius following IBDV challenge. Representative images of hematoxylin and eosin (**H&E**)-stained bursa sections from chickens in the indicated experimental groups at 3 days post-challenge. The top row shows groups challenged with IBDV GF6; the bottom row shows groups challenged with IBDV BC6/85. Scale bar = 50 μm.

Histopathological examination at 3 dpc revealed that vaccinated groups and PBS controls showed minimal or no histopathological changes following challenge. In NDV-challenged controls, severe lesions were observed across multiple tissues (Fig. 8), including tracheal epithelial damage, splenic lymphoid depletion, extensive pulmonary damage (alveolar dilation, septal disruption, cystic spaces, degeneration, necrosis), significant renal congestion, and duodenal villous atrophy with epithelial sloughing and marked lymphocytic infiltration. In IBDV-challenged controls, the bursa of Fabricius exhibited marked lymphoid depletion after challenge with either GF6 or BC6/85 strains (Fig. 9).

### Lower viral loads in vaccinated chickens

**Fig. 10:**
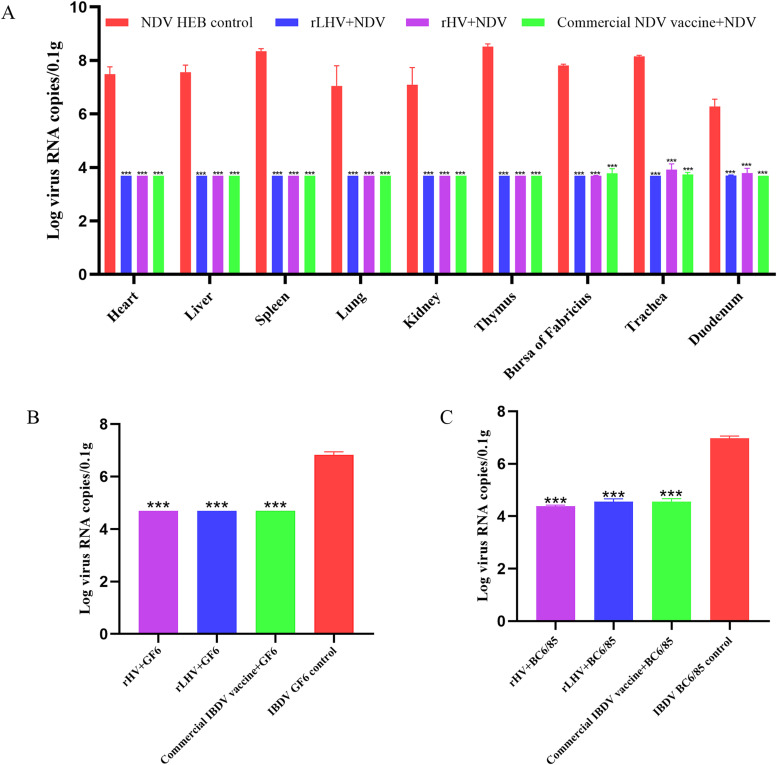
Viral loads were determined by RT-qPCR and expressed as log viral RNA copies per 0.1 g of tissue. (A) NDV viral loads in various tissues (heart, liver, spleen, lung, kidney, thymus, bursa of Fabricius, trachea, duodenum) of chickens challenged with NDV (HEB strain). (B) IBDV viral loads in the bursa of Fabricius of chickens challenged with the IBDV GF6 strain. (C) IBDV viral loads in the bursa of Fabricius of chickens challenged with the IBDV BC6/85 strain. Data are presented as mean ± SEM. Asterisks indicate statistically significant differences compared to the respective unvaccinated control group (***P < 0.001).

Vaccination significantly reduced tissue viral loads compared to unvaccinated controls (***P < 0.001), as quantified by RT-qPCR (Fig. 10). For the NDV challenge, substantially lower viral loads were found across all examined tissues (heart, liver, spleen, lung, kidney, thymus, bursa of Fabricius, trachea, and duodenum) in chickens vaccinated with rHV, rLHV, or the commercial NDV vaccine, relative to the high levels detected in the NDV HEB control group (Fig. 10A). This protective effect was also evident following IBDV challenge, where vaccination with rHV, rLHV, or the commercial IBDV vaccine resulted in significantly reduced viral loads in the bursa of Fabricius compared to the respective high levels found in control groups challenged with either GF6 (Fig. 10B) or BC6/85 (Fig. 10C).

## Discussion

The emergence of nVarIBDV strains, characterized by significant antigenic drift, poses substantial challenges to poultry health worldwide. These evolving strains render many conventional vaccines ineffective, contributing to persistent disease outbreaks and considerable economic losses (Xu et al., 2020; Jiang et al., 2021). Limitations of current IBDV vaccination strategies include antigenic mismatches with circulating field strains, inadequate cross-protection, and difficulties keeping pace with rapid viral evolution. Traditional IBDV vaccines may target epitopes that are altered in nVarIBDV strains like SHG19, highlighting the need for vaccines reflecting current epidemiology. Addressing this, our study utilized the VP2 gene from the nVarIBDV SHG19 strain. As the major protective antigen of IBDV containing critical neutralizing epitopes, VP2 can be tailored to contemporary strains, potentially enhancing protection against field variants and underscoring the need for adaptable vaccine strategies.

NDV vaccines have long been a cornerstone of poultry disease control, with live attenuated strains like LaSota and B1 widely used to protect against ND due to their robust immunogenicity and ability to induce both systemic and mucosal immunity (Dimitrov et al., 2017). However, the emergence of genetically diverse NDV strains, particularly genotype VII, has exposed limitations in current vaccines, including reduced efficacy against antigenically mismatched field isolates (Dewidar et al., 2022). This evolving landscape necessitates next-generation NDV vaccines with enhanced safety, broader antigenic coverage, and ideally, the capacity to address concurrent pathogens like IBDV. NDV itself is a versatile vaccine vector candidate owing to its established safety profile, robust replication, genomic stability, and amenability to genetic manipulation via reverse genetics for expressing foreign antigens (Zhao et al., 2015). We therefore chose NDV as the vector backbone in this study, aiming to leverage its properties to create a bivalent vaccine protecting against both NDV and IBDV, thereby potentially streamlining vaccination protocols and reducing costs.

Our study utilized the VP2 gene from the nVarIBDV SHG19 strain as the target immunogen, incorporating it into the NDV genome to develop a bivalent recombinant vaccine. The VP2 gene was strategically inserted between the P and M genes of the NDV genome, a site previously shown to support stable foreign gene expression without compromising NDV replication (Zhao and Peeters, 2003; Zhao et al., 2015). This design aimed to ensure efficient VP2 expression while preserving the vector’s immunogenicity and replication capacity, enabling simultaneous protection against NDV and IBDV.

The NDV vector allows for the flexible replacement of protective antigens with updated vaccine cassettes derived from currently circulating field strains(Kim and Samal, 2019). Once the sequence of a relevant VP2 gene from a new field isolate is identified, the corresponding gene cassette can be synthesized and cloned into the established NDV vector plasmid, replacing the existing VP2 sequence. Following confirmation of the modified plasmid, the rescue of the updated recombinant virus can potentially be achieved relatively rapidly, potentially within several weeks. This inherent flexibility allows for a faster response to evolving IBDV strains compared to traditional vaccine development approaches.

This study successfully generated two recombinant NDVs, rHV and rLHV, incorporating strategic genetic modifications to optimize safety, immunogenicity, and antigenic relevance. The rHV construct featured nucleotide mutations in the F gene resulting in a modified cleavage site. These mutations convert the multibasic amino acid sequence characteristic of virulent strains (like HEB) to a monobasic sequence typical for lentogenic strains (like LaSota). This established attenuation strategy reduces virulence while maintaining replication competence, ensuring vaccine safety (Le et al., 1988; Xiao et al., 2012). In contrast, rLHV utilized the safe LaSota backbone but incorporated the F and HN genes from the genotype VII HEB strain. This approach aimed to combine LaSota’s proven safety profile with the enhanced antigenic match provided by the surface glycoproteins of currently circulating genotype VII NDV strains, thereby improving protection against field isolates (Sultan et al., 2022). By integrating the VP2 gene into these vectors, the resulting recombinant viruses represented a bivalent vaccine platform that addresses both NDV and IBDV challenges, demonstrating the adaptability of NDV for developing safe and efficacious poultry vaccines through targeted genetic manipulation.

Both recombinant viruses, rHV and rLHV, demonstrated the potential for improved protection against NDV and IBDV. Both elicited robust humoral immune responses against both NDV and IBDV, with HI and ELISA titers comparable to those induced by commercial vaccines (Fig. 4), and their protective efficacy was notable. Although rHV induced lower HI titers than the commercial NDV vaccine, it exhibited superior control of NDV viral shedding and significantly reduced NDV-associated pathology (Table 2, Fig. 6, Fig. 8). This highlights the likely contribution of robust cellular and mucosal immune responses, a possibility supported by previous studies (Ji et al., 2018). Future studies should focus on detailed immunological profiling to further elucidate the contribution of cellular and mucosal immunity to the observed protection. The rLHV also demonstrated a clear advantage in reducing NDV viral shedding compared to the commercial NDV vaccine, likely due to improved antigenic matching to circulating genotype VII strains (Roohani et al., 2015).

Furthermore, both rHV and rLHV also significantly reduced IBDV-induced bursal pathology (Fig. 7, Fig. 9), further demonstrating their efficacy as bivalent vaccines. Following challenge with either IBDV GF6 (a novel variant strain) or BC6/85 (a standard challenge strain), both recombinant viruses significantly reduced viral shedding and bursal viral load compared to their respective IBDV challenge control groups. Notably, the number of PCR-positive cloacal swabs from the recombinant virus groups was slightly lower than from the commercial IBDV vaccine groups (Table 3, Table 4). Furthermore, viral loads in the bursae of Fabricius were significantly reduced in all vaccinated groups compared to the control groups (Fig. 10B and 10C). These results indicate that both rHV and rLHV provide effective protection against both the nVarIBDV strain GF6 and the standard challenge strain BC6/85. Although slightly lower antibody titers were observed with the recombinant NDV compared to the commercial vaccines, the comparable or superior protective efficacy strongly supports the importance of developing vaccines with optimized antigenic matches to circulating strains, particularly the prevalent nVarIBDV strains, while also considering the potential for future antigenic drift. The use of two distinct IBDV challenge strains – GF6, representing the emerging threat of nVarIBDV, and BC6/85, a standard strain for evaluating vaccine efficacy – allowed for a more comprehensive evaluation of the vaccine's protective efficacy against a broader range of IBDV challenges. This approach mimicked the diverse IBDV strains encountered in the field, providing a more realistic assessment of the vaccine's potential for practical application.

## Conclusion

This study successfully developed two recombinant NDVs expressing the VP2 protein of IBDV, rHV and rLHV, which demonstrated robust dual protection against NDV and IBDV, including both the nVarIBDV GF6 strain and the standard IBDV BC6/85 strain. The recombinant vaccines elicited strong humoral immune responses, significantly reduced viral shedding, and minimized pathological changes in vaccinated chickens following challenges with both NDV and IBDV. These findings highlight the potential of NDV-based bivalent vaccines as effective tools for controlling multiple poultry pathogens, paving the way for further advancements in multivalent vaccine design and implementation.

## Ethics statement

The care and use of animals in this study adhered to all relevant international and national guidelines. Approval for animal experiments was obtained from the Committee on the Ethics of Animal Experiments at Northeast Agricultural University, Harbin, China (2016 NEFU-315, 13 April 2017).

## Declaration of competing interest

The authors declare that they have no known competing financial interests or personal relationships that could have appeared to influence the work reported in this paper.

## Appendix Supplementary materials

Image, application 1
