# Supplementary material for: Development and evaluation of immunogenicity and protective efficacy of two recombinant attenuated newcastle disease viruses expressing the VP2 protein of infectious bursal disease virus
Source: Poult Sci. 2025 May 8;104(7):105253. doi: 10.1016/j.psj.2025.105253 (PMC12138426; doi:10.1016/j.psj.2025.105253)
Supplement: Supplementary file 1 [file mmc1.docx]

**Supplementary Table 1.** Primer sequences used to amplify the genes of IBDV VP2 and NP/P/L.

| Primer Name | Primer sequences (5’to3’) | Fragment |
| --- | --- | --- |
| H-VP2-F | TGCAAATCCACGCTT**GTTTAAA**CTTAAGAAAAAAT | VP2 |
|  | ACGGGTAGAAGCCACCATGACCAACCTGCAGGAC | (HEB) |
| H-VP2-R | GAGGGCTGTTGTTTT**GTTTAAA**CTTATCTCCTTAG |  |
|  | GGCCCTGATAAT |  |
| L-VP2-F | CCGCAGACCCAAGGT**GTTTAAA**CTTAAGAAAAAA | VP2 |
|  | TACGGGTAGAAGCCACCATGACCAACCTGCAGGA | (LaSota) |
| L-VP2-R | AGGATTGCCGCTTGG**GTTTAAA**CTTATCTCCTTAG |  |
|  | GGCCCTGATAAT |  |
| H-NP-F | ataggctagcctcga**gaattc**cGCCACCATGTCGTCTGTCT | NP (HEB) |
| H-NP-R | gccgcccgggtcgac**tctaga**TTATCAGTATCCCCAATCAGTGTCG |  |
| H-P-F | ataggctagcctcga**gaattc**cGCCACCATGGCCACTTT | P (HEB) |
| H-P-R | gccgcccgggtcgac**tctaga**TTATCAACCATTCAGCGCAAGG |  |
| H-L-F | ataggctagcctcga**gaattc**cGCCACCATGGCGGGCTC | L (HEB) |
| H-L-R | gccgcccgggtcgac**tctaga**CTATTAAGAGTCATTATTACTGTAA |  |
| L-NP-F | ataggctagcctcga**gaattc**cGCCACCATGTCTTCCGTATTTGATG | NP (LaSota) |
| L-NP-R | gccgcccgggtcgac**tctaga**TTATCAATACCCCCAGTCGGTG |  |
| L-P-F | ataggctagcctcga**gaattc**cGCCACCATGGCCACCTTTACAGAT | P (LaSota) |
| L-P-R | gccgcccgggtcgac**tctaga**CTATTAGCCATTTAGAGCAAGGCG |  |
| L-L-F | ataggctagcctcga**gaattc**cGCCACCATGGCGAGCTCCGGTCCT | L (LaSota) |
| L-L-R | gccgcccgggtcgac**tctaga**CTATTAAGAGTCACAGTTACTGTA |  |

## Bold text indicates restriction endonuclease sites, lowercase letters indicate sequences homologous to the pCI vector, and underlined sequences indicate those homologous to the pCI-aH (HEB backbone) or the pCI-L-H(aF/HN) (LaSota backbone).

**Supplementary Table 2.** Primers for RT-qPCR quantification of viral loads in organs.

| Primer Name | Primer sequences (5’to3’) | Fragment |
| --- | --- | --- |
| HEB-F | ACAGGGTCAATCATAGTCA | 181 bp |
| HEB-R | AGCGTTTTTGTCTCCTTC |  |
| GF6-F | TGGATGCGACAGATAGAA | 191 bp |
| GF6-R | CTGGAACCTGGAGAAACA |  |
| BC6/85-F | TGGGACAGGCTACCAGACTT | 170 bp |
| BC6/85-R | CCCTTGTGAGTGGTACCCAG |  |

## Primer sequences used for RT-qPCR to quantify viral loads in organ samples. The HEB primers target the F gene, while the GF6 and BC6/85 primers target the VP1 gene.


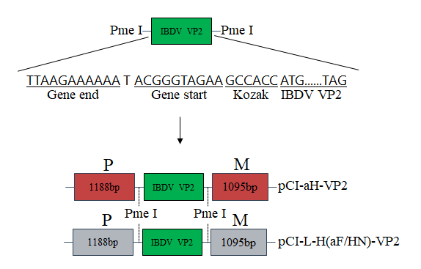


**Supplementary Figure 1.** Construction of IBDV VP2 expression cassette and insertion into NDV backbones. An expression cassette containing the IBDV VP2 gene, flanked by *Pme* I sites and incorporating NDV gene end, gene start, and Kozak signals, was generated. This cassette was inserted via PmeI sites into the P/M gene junction of two modified NDV backbones: the HEB-derived pCI-aH backbone (yielding pCI-aH-VP2) and the LaSota-derived backbone containing HEB F/HN genes, pCI-L-H(aF/HN) (yielding pCI-L-H(aF/HN)-VP2).


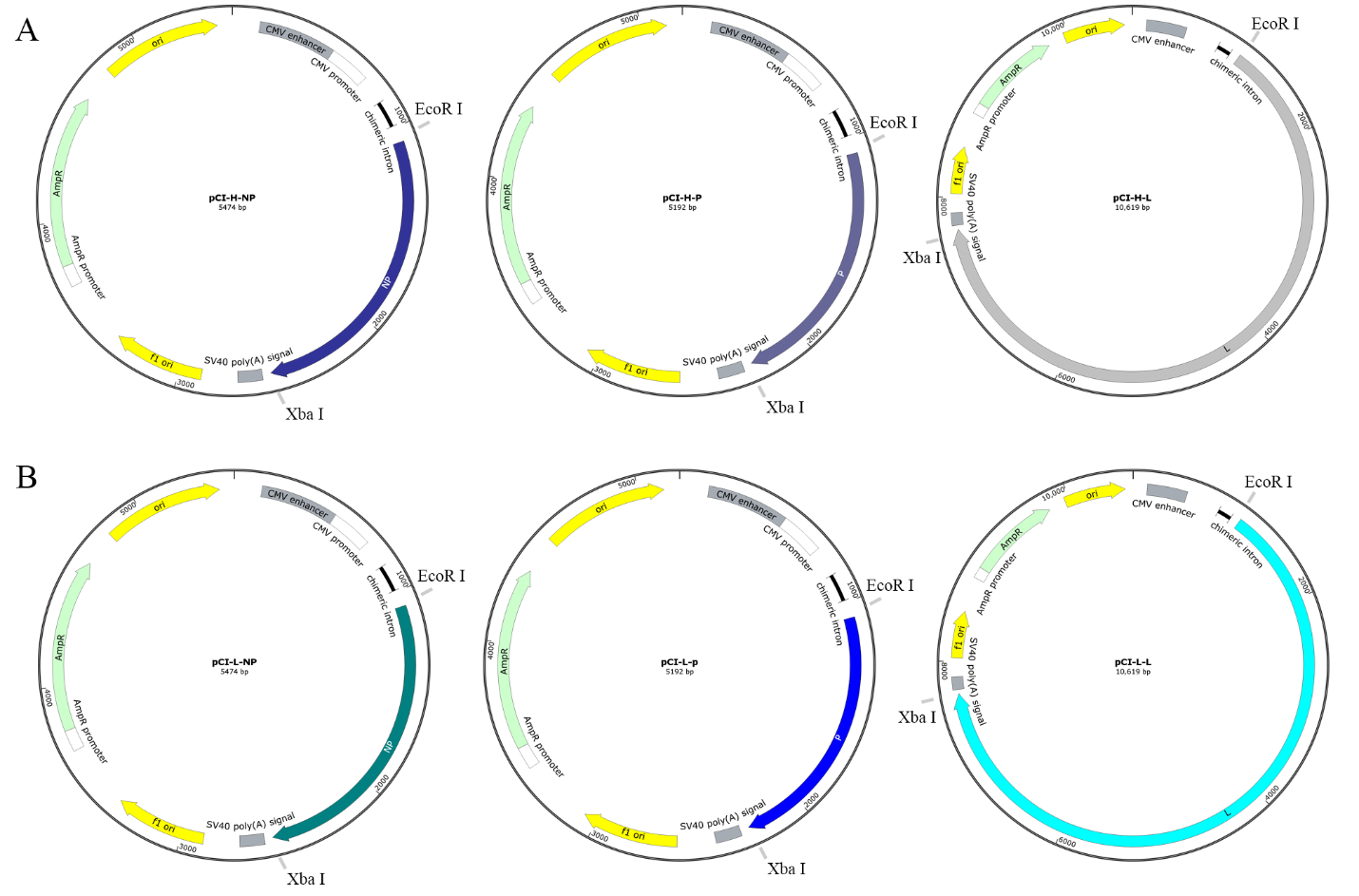


**Supplementary Figure 2.** Plasmid maps of NDV helper constructs. The figure displays the maps for two sets of helper plasmids constructed for recombinant virus rescue. (A) Plasmids designed to express the NP, P, and L proteins derived from the genotype VII HEB strain. The respective genes (NP, P, L) were inserted into the pCI eukaryotic expression vector between the *EcoR* I and *Xba* I restriction sites, creating pCI-H-NP, pCI-H-P, and pCI-H-L. (B) Plasmids designed to express the corresponding NP, P, and L proteins derived from the LaSota strain. Similarly, the LaSota NP, P, and L genes were cloned into the pCI vector between the *EcoR* I and *Xba* I sites, generating pCI-L-NP, pCI-L-P, and pCI-L-L.
